# Application of carbon from pomegranate husk for the removal of ibuprofen, cadmium and methylene blue from water

**DOI:** 10.1016/j.heliyon.2023.e20268

**Published:** 2023-09-19

**Authors:** N.D. Shooto

**Affiliations:** Adsorption Laboratory, Natural Sciences Department, Vaal University of Technology, P.O. Box X021, Vanderbijlpark, 1900, South Africa

**Keywords:** Pomegranate husk, Ibuprofen, Cadmium, Methylene blue, Sorption, Carbon

## Abstract

The presence of pharmaceutical products, dyes, and toxic metal ions in water is a major problem worldwide. This work developed low-cost pomegranate-based materials to uptake ibuprofen, cadmium and methylene blue from water. Pomegranate husks (PPH) were carbonized at 400 °C to form carbonized pomegranate husk (CPH), and nanoparticles were loaded into the carbon surface (NPH) by co-precipitation. SEM micrographs showed that the morphology of carbon was highly porous compared to pristine pomegranate husk. The data for BET revealed that CPH and NPH, had about a 20-fold increase in surface area of 142 m^2^/g and 190 m^2^/g respectively compared with 9.27 m^2^/g for PPH. The composites exhibited larger pore sizes and volumes. TEM images confirmed the loading of nanoparticles. The FTIR results showed that the materials had on their surface oxygenated groups such as –OH, –C]O, –COC and other groups like –NH and –C]C which are anticipated to play an essential role in the sorption of the pollutants. It was found that removal efficiency increased when there was a progressive increase in pollutant concentration for all adsorbents. The best pH value of the solution for the sorption processes was pH 8. The recorded adsorption capacities at pH 8 for Cd(II), IBU and MB were 92.85, 39.77 and 95.89 mg/g for NPH, 72.60, 32.58 and 80.59 mg/g for CPH and 32.78, 16.12 and 40.79 mg/g for PPH. Contact time studies showed three sorption steps. Step 1: rapid increase at the initial stage. Step 2: marginal uptake. Step 3: plateau. The trends indicated that sorption was influenced by temperature variation. The data for the thermodynamic parameter △*H*^*o*^ suggest that all the sorption processes were endothermic; the obtained positive values indicate this. The △*H*^*o*^ for PPH was between (64.33–69.08 kJ/mol), 82.84–86.03 kJ/mol for CPH and 87.17–88.96 kJ/mol for NPH. For PPH, molecular interactions were physisorption, and chemisorption for CPH and NPH. The △*S*^*o*^ has positive values, showing increased freedom during the sorption. The adsorbents followed PSO based on uptake processes involving syngenetic mechanisms.

## 1 Introduction

Chemical and bacterial contaminants in drinking water have affected multitudes of people worldwide [1,2]. It was reported that in some areas in Bangladesh, more than 20 million people are ingesting water containing pollutants having higher concentrations than the standard set out by the local government and World Health Organisation (WHO) [3]. Ibuprofen (IBU) is a drug used to relieve pain, fever and inflammation [4] and is manufactured in large quantities due to its many uses. It enters water systems through human and animal excretions and improper disposal of medicines containing IBU or its metabolites by hospitals and pharmaceutical industries worldwide [5,6]. IBU in drinking water has adverse effects on living organisms with long term exposure [7]. Therefore, the elimination of IBU from water is necessary.

Cadmium ions (Cd(II)) originate from chemical industries such as mining, leather tanning, cadmium (Cd)-nickel (Ni) batteries and pesticides manufacturers [8]. Cd(II) in drinking water is of concern because it is toxic, non-metabolized by living organisms and is not biodegradable. Over time it accumulates in the environment, water bodies and living organisms. Furthermore, even at low concentrations, Cd(II) ions cause serious complications to human beings, such as permanent damage to the brain, long-term kidney and lung problems, and irreversible damage to the skeletal system [9]. Methylene blue (MB) is a toxic synthetic dye at high concentrations [10,11]. It is widely used in textile and dying industries for colouring fabrics [12]. And is frequently disposed into water bodies [13]. The toxicity of MB causes skin irritation and cancer, abnormal heart rates, vomiting, neuronal apoptosis, and inflammation of the leptomeninges [14,15].

Ternes et al. [16] reported that conventional methods, including oxidation, flocculation and coagulation, ion exchange, phytoremediation and sedimentation, are ineffective in eliminating the abovementioned substances from wastewater. On the other hand, the non-conventional method of adsorption is more effective and efficient [[17], [18], [19], [20], [21]]. The presence of benzene rings and functional groups on the structures of IBU and MB enhances the ability of carbonaceous material derived from agricultural waste to adsorb these substances from water [22,23]. The properties of carbonaceous materials can be adjusted by treatment with solutions of oxidizing agents (KMnO_4_, NaOCl, HNO_3_, H_2_SO_4_, H_2_O_2_, H_3_PO_4_ etc) [8,[24], [25], [26]]. The reactions introduce oxygen-rich functional groups and alter the materials textural properties and surface roughness, greatly improving the sorption capacity.

Agricultural waste materials include plant-based by-products typically disposed of in the environment after use [27]. This work will investigate pomegranate (*Punica granatum* L) husks to remove the earlier-mentioned water pollutants. The annual Pomegranate production is about 300 million tons, with Asia contributing about 195 million tons and Tunisia producing 71 [28]. Husk makes up about 30% of the fruit and is discarded as waste. Worldwide, pomegranate industries (juice and jam) and supermarkets generate tons of husk waste by-products which pollute and cause a nuisance in the environment [29]. Several studies investigated the potential of pomegranate-based materials in water treatment Ghadi [30] prepared and utilized carbon from pomegranate husks for the uptake of Cu(II) from water, while Ben-Ali [31] used untreated pomegranate husks to remove Cu(II) ions. The removal of remazol brilliant blue R dye with carbon from pomegranate husk was reported by Ref. [32]. Rashbari [33] prepared pomegranate-nanocomposite materials with a large surface area to remove cephalexin. Magnetized carbon from pomegranate husk was used to remove 4-chlorophenol [34]. Ahmad [35] carried out the experiments for removing MB using carbon derived from pomegranate husk. A blend of black clay and pomegranate husk was used in adsorption of Cr(VI) by Ramadan [36], while Suhaimi [37] investigated the mixture of carbons from carrot pulp and pomegranate peel to remove crystal violet dye. Akkari [38] reported on the uptake of basic red 46 dye using carbonized pomegranate husk.

The aim of this work was to explore pomegranate husk as a cost effective and alternative material for wastewater treatment. This is the first report on the sorption of ibuprofen and cadmium using pomegranate-based materials. Few studies have reported on removing methylene blue using pomegranate husks. Pomegranate husks (PPH) were heated at 400 °C to produce their carbonized form (CPH), and Fe(II)–Fe(III) nanoparticles were loaded onto the carbon by co-precipitation to prepare its nanoparticle derivative (NPH). The effectiveness of the materials (PPH, CPH and NPH) were utilized for the sorption of pharmaceutical product, dye, and metal ions from water. This work generated data for variables such as the effect of initial concentration, solution pH, the temperature of the system, and contact time that were incorporated into non-linear models of isotherms and kinetics.

## 2 Materials and methods

### 2.1 Materials

The pomegranate husks were collected from a fruit and vegetable store in Vanderbijlpark, South Africa. The chemicals; cadmium nitrate (Cd(NO_3_)_2_) ≥99.9%, hydrochloric acid (HCl) ≥37.0%, methylene blue (C_16_H_18_ClN_3_S) abbreviated (MB) ≥97.0%, ammonium hydroxide (NH_4_OH) ≥28.0% sodium hydroxide (NaOH) ≥98.0% and ibuprofen (C_13_H_18_O_2_) abbreviated (IBU) ≥98.0%, were procured from Sigma Aldrich (Johannesburg, South Africa).

### 2.2 Preparation of pomegranate sorbents

#### 2.2.1 Pristine pomegranate husks

A Lasec portable pulveriser was used to grind the dry husk into a fine powder. Material with particle a size of 0.8–1.0 mm was collected using a sieve. The material was soaked in 500 mL deionized water and stirred. Then, the material was dried at 35 °C for 12 h and designated as pristine pomegranate husks (PPH).

#### 2.2.2 Carbonized pomegranate husks

PPH was carbonized in a furnace at 400 °C in a nitrogen atmosphere. The sample was heated for 60 min and removed from the furnace when cool. The carbonaceous sample was designated carbon from pomegranate husks (CPH).

#### 2.2.3 Pomegranate composite

The surface of the CPH was loaded with Fe(II)/Fe(III) based nanoparticles, following the method reported by Ref. [39]. CHS (10 g) was transferred to a vial containing 100 mL de-ionized water. Five grams of Fe(NO_3_)_3_ and Fe(NO_3_)_2_ were added to the solution. The pH of the solution was made alkaline with 10 mL of NH_4_OH solution. The reaction was stopped after 3 h. The solution was decanted, and the sample was kept in the oven for 12 h. The reaction mixture was stirred throughout the experiment.

## 3 Experiments

### 3.1 Batch sorption experiments

The experiments were conducted as follows: the initial concentration effect (20–100 mg/L), solution pH between (2−10), contact time at 5–360 min and temperature effect between 288 and 308 K.

### 3.2 Sorption data management

The sorption capacity, *q*_*e*_ (mg/g) of the materials, was determined using (eq (1)).

(1) $$q_{e}=\frac{\left(C_{o}\;–\;C_{e}\right)\;V}{W}$$

### 3.3 Analytical methods

SEM-EXD (Thermo Fisher, Waltham, MA) characterized the exterior morphology and chemical content of the adsorbents. TEM (Joel Ltd., Tokyo, Japan) analyzed the interior morphology of the adsorbents and the nanoparticles. XRD (Shimadzu Corporation, Kyoto, Japan) determined the phase purity of the materials. FTIR (PerkinElmer, Waltham, MA) identified the functional groups on the surface of the materials. BET (Georgia Corporation, Peachtree Corners, GA) was employed to determine the physicochemical properties of the materials. ICP (Thermo Fisher Scientific) and UV–Visible (Thermo Fisher Scientific) confirmed the concentration of Cd(II), MB and IBU.

## 4 Results and discussion

### 4.1 Physicochemical properties of pomegranate materials

Table 1 contains some of the physicochemical properties of the adsorbents. The BET data indicated that CPH and NPH, had about a 20-fold increase in surface area of 142 m^2^/g and 190 m^2^/g respectively, compared with 9.27 m^2^/g for PPH. The composites had larger pores and volumes. This was attributed to the removal of volatile components during carbonization [40,41]. The pH_(PZC)_ for PPH was 5.2, while that for CPH and NPH were 4.8 and 5.9, respectively. CPH became more acidic by a slight shift to pH 4.8. This was attributed to the formation of oxygen-containing functional groups on the surface of the material. NPH recorded a slight increase to pH 5.9 due to the deposition of Fe(II)–Fe(III) nanoparticles.

**Table 1:** Physicochemical properties of PPH, CPH and NPH.

Adsorbent	BET			pH_(PZC)_
	Surface area (m^2^/g)	Pore size (nm)	Pore width (nm)	
PPH	9.270	0.0189	0.0291	5.2
CPH	142.0	0.0951	0.0829	4.8
NPH	190.0	0.0883	0.0790	5.9

### 4.2 Characterization

#### 4.2.1 FTIR

**Fig. 1:**
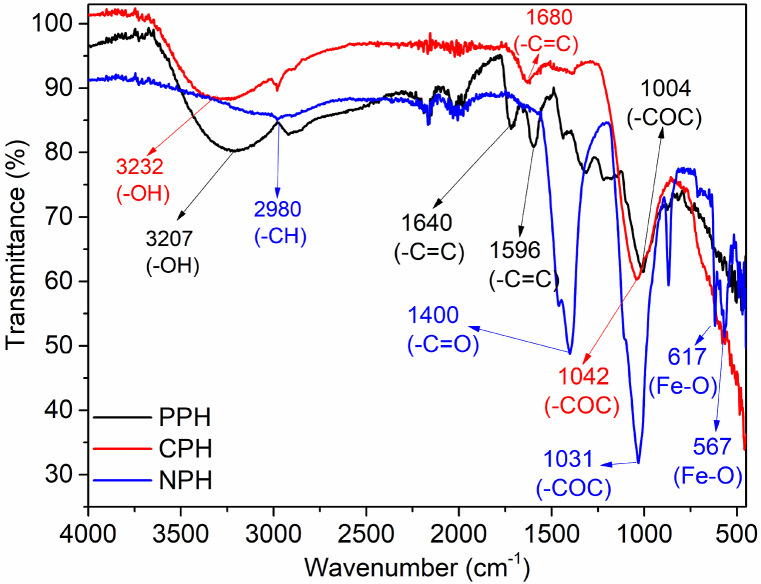
FTIR spectra for pomegranate-based adsorbents.

The FTIR data validated the groups incorporated on the surface of the pomegranate sorbents (Fig. 1). The broad peaks at wavenumber 3207 and 3232 cm^−1^ for PPH and CPH are ascribed to the overlapping vibrations of the hydroxyl and amine groups [42,43]. A peak at 2980 cm^−1^ for CPH and NPH is attributed to symmetric stretching of the –CH group [44]. PPH and CPH recorded peaks at wavenumber 1596, 1640 and 1680 cm^−1^ assigned to aromatic rings (–C]C) [[45], [46], [47]]. All the samples had a carboxyl (–C]O) group at wavenumber 1400 cm^−1^, while the cellulosic (–COC) group was present at 1004, 1031 and 1042 cm^−1^ for PPH, NPH and CPH [48]. The peaks for (Fe–O) nanoparticles were recorded at wavenumber 617 and 567 cm^−1^ [49].

#### 4.2.2 SEM

**Fig. 2:**
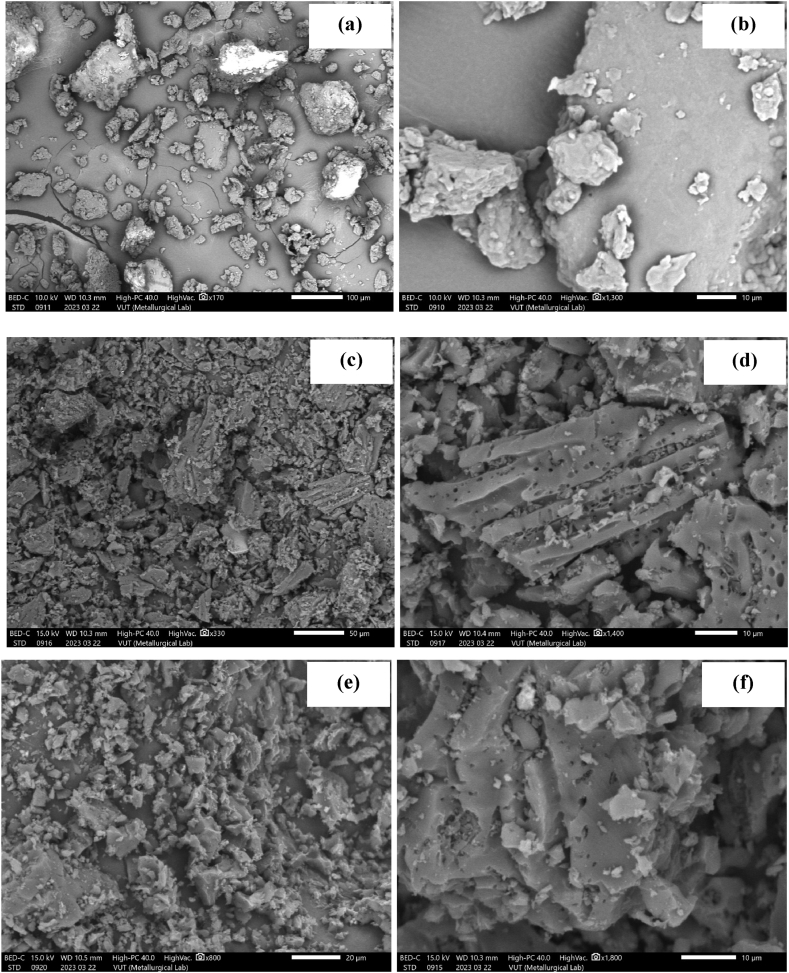
SEM images for PPH (a–b), CPH (c–d) and NPH (e–f).

SEM images (Fig. 2a–f) were investigated to assess the material's morphology. The images for PPH, CPH and CPH in Fig. 2a, c and e reveal that the material was composed of random-sized particles of variable sizes and amorphous shapes. The surface of PPH (Fig. 2b) was heterogenous, while CPH (Fig. 2d) and CPH (Fig. 2f) had unique openings and pores. This was attributed to the volatilization of some components during the carbonization process [40,41,50].

#### 4.2.3 EDX

**Fig. 3:**
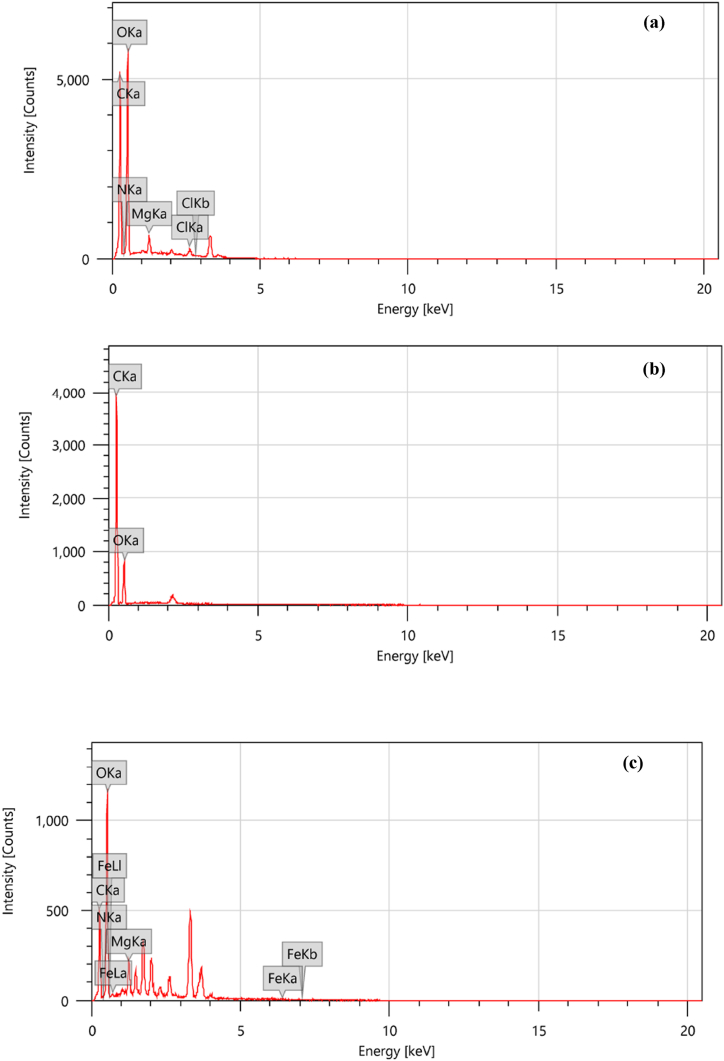
EDX spectra for PPH (a), CPH (b) and NHP (c).

An adsorbent's chemical constituents mainly govern the adsorptive behaviour of the material. Therefore, the elemental analyses of PPH, CPH and NPH were investigated, and data is presented in Fig. 3a–c. All the materials contained a high content of the elements, carbon (C) and oxygen (O). PPH had additional elements in trace amounts, such as nitrogen (N), magnesium (Mg) and chlorine (Cl). These are naturally occurring plant-based materials [Khalil et al., 2022; Taheri et al., 2022]. NPH had iron (Fe) attributed to the loaded Fe(II)–Fe(III) nanoparticles.

#### 4.2.4 XRD

**Fig. 4:**
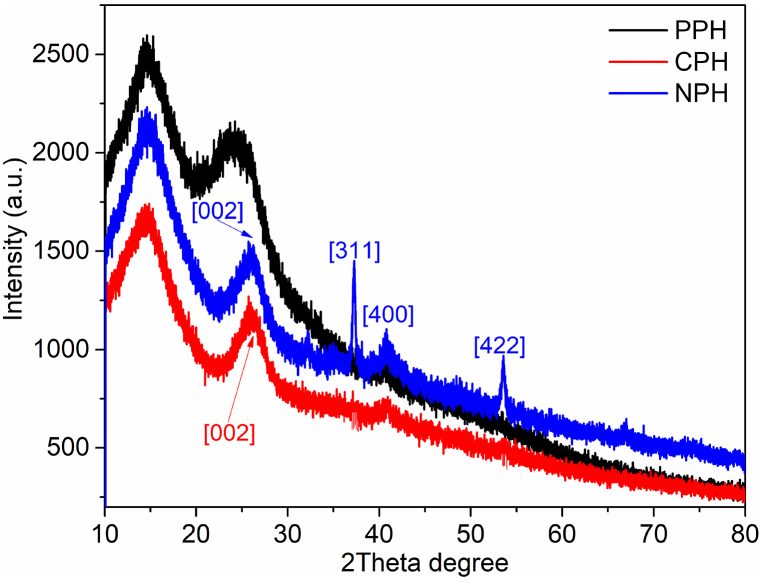
XRD spectra for pomegranate material.

The crystallinity of the pomegranate-based materials was determined using diffraction spectra as shown in Fig. 4. PPH showed two peaks at 2theta = 15 and 24° characteristic of the crystalline structure of cellulose [51]. The diffraction spectra for CPH and NPH showed a peak assigned to cellulose at 2theta = 15°. This suggests that there were traces of cellulose in the samples. CPH and NPH showed a new peak at 2theta = 27° indexed (002) attributed to amorphous carbon. The pattern for NPH showed unique peaks at 2theta = 36.7, 42.7 and 53.5° corresponding to planes (311), (400) and (422), respectively, indicating Fe_3_O_4_ phase [52] (JCPDS card number: 75–0033).

#### 4.2.5 TEM

**Fig. 5:**
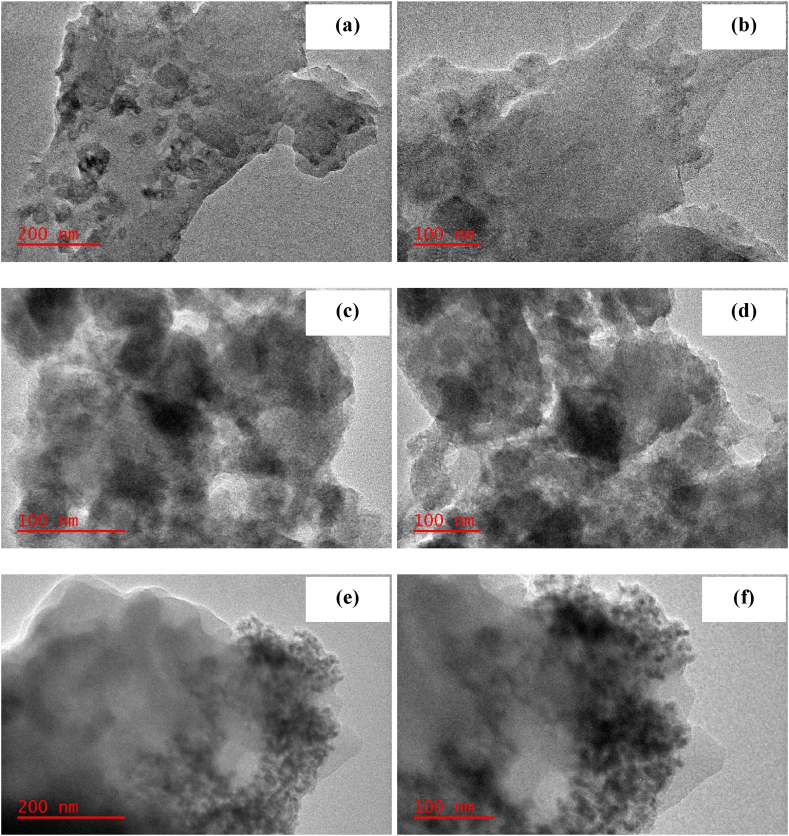
TEM images for PPH (a–b), CPH (c–d) and NHP (e–f).

TEM images were scrutinized to investigate the morphology of PPH, CPH and NPH (Fig. 5a–f). The images revealed that the surfaces of PPH (Fig. 5a–b) and CPH (Fig. 5c–d) were uniform. However, the images for NPH (Fig. 5e–f) show the growth of particles on the material confirming the deposition of Fe(II)–Fe(III) nanoparticles on the surface of the carbon.

### 4.3 Sorption data

#### 4.3.1 Initial concentration effect

**Fig. 6:**
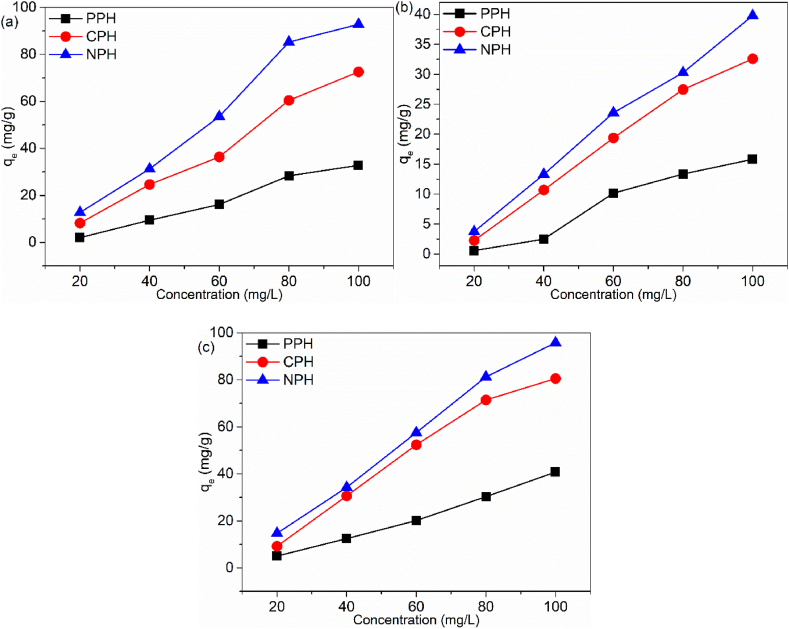
Initial concentration effect on (a) Cd(II), (b) IBU and (c) MB.

The influence of the initial concentration on the removal efficiency was evaluated (Fig. 6a–c). The trends showed that initial concentration plays a significant role in sorption processes. The removal efficiency increased with a progressive increase in pollutant concentrations. The lowest adsorption capacity was recorded for solutions of 20 mg/L while the highest removal occurred for solution of 100 mg/L. This result is due to the overcoming of the forces hindering sorption when the initial concentration was gradually increased from 20 to 100 mg/L and resulted in1 enhanced adsorptive driving forces. It was observed that the NPH was better adsorbent than CPH and PPH. The highest adsorption capacities for Cd(II), IBU and MB by the materials were 92.80, 39.88 and 95.91 mg/g for NPH, 72.59, 32.55 and 80.58 mg/g for CPH and 32.81, 15.80 and 40.83 mg/g for PPH. This was facilitated by π-π bonding, hydrogen bonding, Yoshida hydrogen bonding and electrostatic interactions between the adsorbed pollutants on the adsorbent's surfaces and the pollutants in the solution. In addition, diffusion of the adsorbed contaminants on the external surface to the pores occurred [53,54].

#### 4.3.2 Contact time effect

**Fig. 7:**
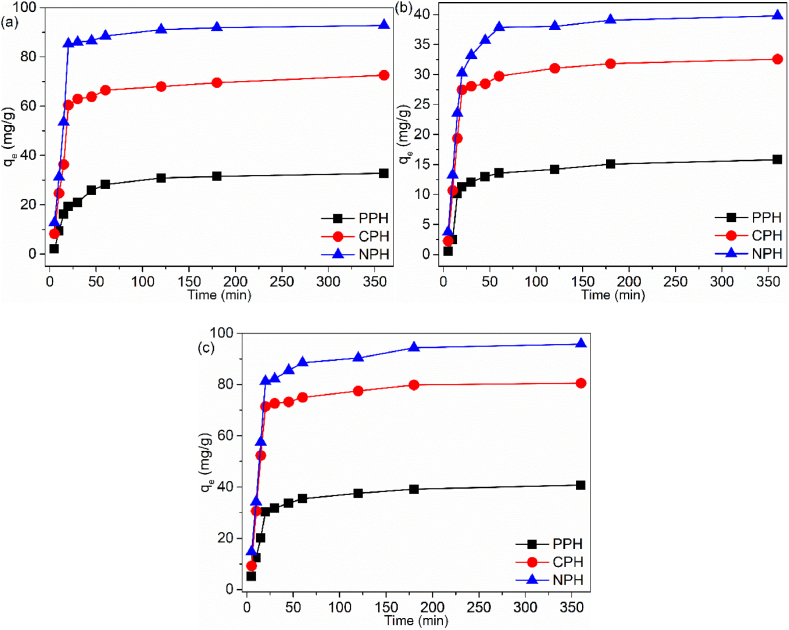
Contact time effect on (a) Cd(II), (b) IBU and (c) MB.

Contact time was determined to ascertain the rate of uptake efficiencies at different intervals. (Fig. 7a–c). The rates of pollutant uptake were investigated between 5 and 360 min. Similar trends were found for the adsorbents, and the removal efficiency had three steps. Step 1: rapid increase at the initial stage. Step 2: marginal uptake. Step 3: plateau. This behaviour was observed for other biomaterials [55]. The rapid increase was because of abundant active sites (functional groups, pores, cavities) and a large surface area for binding on the adsorbent's surface [56]. The slow increase was due to diffusion from the exterior to the interior surface. Plateau indicated saturation of the active sites and marked equilibrium. NPH attained the highest rate, followed by CPH and PPH. Hence NPH attained equilibrium for Cd(II) within 20 min, while CPH and PPH took 40 and 60 min respectively. Equilibrium was reached within 60 min by NPH for IBU. On the other hand, CPH and PPH took 120 min. The equilibrium for MB was within 180 min for all the materials.

#### 4.3.3 Solution pH effect

**Fig. 8:**
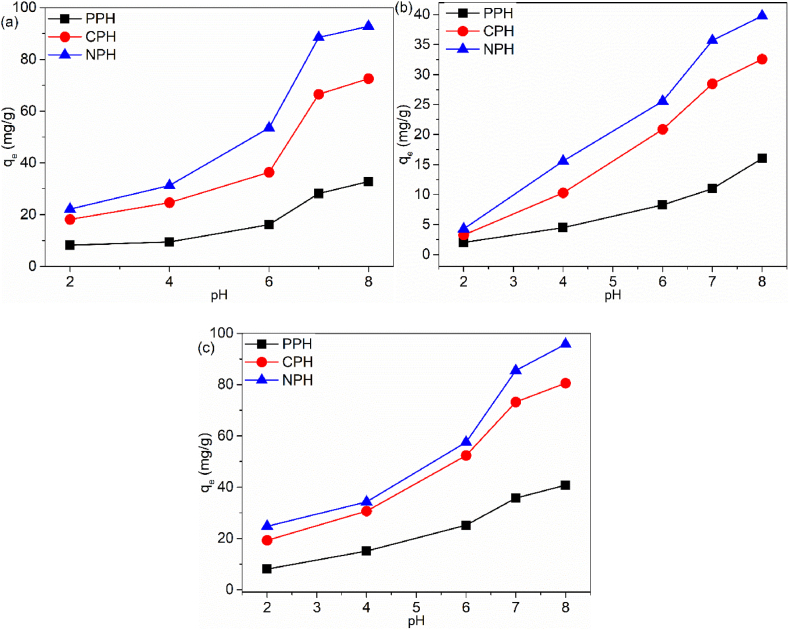
pH effect on (a) Cd(II), (b) IBU and (c) MB.

The adsorbent's surface charge, speciation of the pollutants, and the extent of sorption are influenced by solution pH. This parameter was investigated at a pH range of 2–8 the results are presented in Fig. 8a–c. It was found that an acidic pH solution negatively impacted the removal efficiencies of the pollutants. A solution pH of 2 showed the lowest removal efficiency. At this condition, the electrostatic interaction was highly restricted by the protonated (H^+^ or H_3_O^+^) functional groups on the surface of the adsorbents, and the cationic pollutants sorption occurred through other mechanisms such as van der Waals forces this reduced uptake [57,58]. The recorded adsorption capacities at solution pH 2 were 22.18, 4.277 and 24.74 mg/g for NPH, 18.17, 3.247 and 19.25 mg/g for CPH and 8.248, 2.018 and 8.057 mg/g for PPH for Cd(II), IBU and MB, respectively.

When the solution pH gradually increased (pH 7–8) protonated functional groups and cationic species were reduced in the solution. The adsorbent surface was ionized in an alkaline medium and acquired a negative charge, enhancing the electrostatic interaction between the adsorbents and pollutants [59]. Electrostatic interaction occurred between the adsorbents and pollutants (Cd(II), IBU and MB) through (–OH), (–C]O) and (–NH) groups. Furthermore, the aromatic rings on IBU and MB interacted with the adsorbent through π-π bonds [60]. The best pH value of the solution for the sorption processes was pH 8. The recorded adsorption capacities at solution pH 8 increased to 92.85, 39.77 and 95.89 mg/g for NPH, 72.60, 32.58 and 80.59 mg/g for CPH and 32.78, 16.12 and 40.79 mg/g for PPH for Cd(II), IBU and MB, respectively.

#### 4.3.4 Temperature effect

**Fig. 9:**
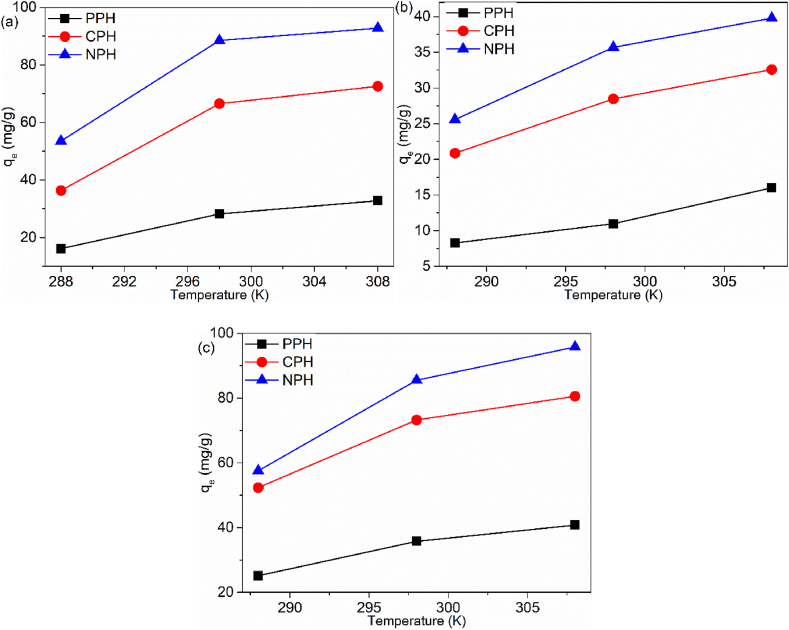
Temperature effect on (a) Cd(II), (b) IBU and (c) MB.

The sorption experiments for the temperature effect were conducted at 288–308 K; the data are shown in Fig. 9a–c. The results showed that sorption was influenced by temperature variation. The uptake efficiency of the pollutants increased when the temperature was gradually elevated. These trends (Fig. 9a–c) revealed that the process was endothermic. Higher removal at elevated reaction is attributed to several factors: (i) lowered activation energy, (ii) increased rate of diffusion, (iii) higher kinetic energy gained which resulted in more collisions between the pollutant and the surface of the adsorbent, and (iv) change in pore volume/size on the surface of the adsorbents which became larger, exposing new active sites [61].

### 4.4 Isotherm, kinetics and thermodynamic studies

#### 4.4.1 Isotherm models

The isotherms were analyzed to better comprehend the uptake behaviour and processes involved in the sorption and to understand the relationship between the adsorbent's equilibrium point and the pollutant's initial concentration in the solution. The Langmuir isotherm [62] is based on the sorption of pollutants on a homogeneous surface by forming a monolayer described by a non-linear form expressed in equation (2). In contrast, the Freundlich model [63] is based on multilayer sorption formation on a heterogeneous adsorbent surface described by a non-linear form expressed in equation (3).

(2) $$q_{e}=\frac{Q_{o}bC_{e}}{1\;{+bC}_{e}}$$

(3) $$q_{m}=k_{f}C_{e}^{1/n}$$

The equilibrium data were integrated into equations of the non-linear isotherms of the Langmuir and Freundlich models, the parameters are presented in Table 2. The suitability of the models was tested by a comparative analysis of the model with regression (*R*^*2*^) closer to unity. The model that complemented the experimental data better was the Freundlich isotherm. The Freundlich model had (*R*^*2*^) values for Cd(II), IBU, and MB by PPH as (0.995, 0.992, 0.990), CPH (0.991, 0.990, 0.990) and NPH (0.998, 0.997 and 0.998). This indicated that sorption had occurred on heterogeneous surfaces with the formation of multilayer adsorption when the concentration of the pollutants was increased [[64], [65], [66]]. According to Siddiqui and Chaudhry [67], the value of the Freundlich constant (*n*) between 0 and 10 indicates that the sorption of pollutants was favorable. Moreover, To et al. [68] and Shikuku and Jemutai-Kimosop [69] suggested that a value of (*n*) below 1 indicates that the uptake had weak adsorptive forces attributed to physisorption. The values obtained for (*n*) for Cd(II), IBU, MB by PPH were (0.027, 0.019, 0.033), CPH (0.057, 0.027, 0.061) and NPH (0.084, 0.032, 0.085) respectively.

**Table 2:** Isotherm models for the pollutants onto pomegranate materials.

Sorption isotherm models	Parameters	PPH			CPH			NPH		
		Cd(II)	IBU	MB	Cd(II)	IBU	MB	Cd(II)	IBU	MB
Langmuir	*Qo (mg/g)*	28.80	11.53	35.44	66.70	27.69	76.50	88.03	35.60	90.14
	*B (L/mol)*	1.791	1.372	1.802	1.826	1.703	1.885	1.919	1.799	1.922
	*R*^*2*^	0.974	0.979	0.978	0.981	0.985	0.987	0.989	0.980	0.988
Freundlich	*K*_*F*_	32.74	15.85	41.09	73.12	34.01	82.14	92.82	40.68	95.80
	*N*	0.027	0.019	0.033	0.057	0.027	0.061	0.084	0.032	0.085
	*R*^*2*^	0.995	0.992	0.990	0.991	0.990	0.990	0.998	0.997	0.998
Experimental	*q*_*e*_*(mg/g)*	32.66	15.71	40.80	72.63	32.58	80.59	92.75	39.80	95.77

#### 4.4.2 Kinetic models

Kinetics were determined to understand the mechanism controlling the adsorption. Kinetics were calculated using non-linear models of pseudo-first-order (PFO) equation (4), pseudo-second-order (PSO) equation (5) and intraparticle diffusion (IPD).

(4) $$q_{e}=q_{t}\left(1-e^{-k_{1}t}\right)$$

(5) $$q_{e}=\frac{1+k_{2}q_{e}t}{k_{2}q_{e}^{2}t}$$

The experimental data for contact time was fitted to non-linear kinetic models. PFO and PSO models established the uptake rate and mechanism(s) shown in Table 3. A comparative analysis for PFO and PSO (*R*^*2*^) values closer to unity (1) was used to predict the best-fitting model. PSO (*R*^*2*^) values were higher than those for PFO, indicating that PSO described the experimental data better. PSO is based on the chemisorption process, which involves syngenetic mechanisms such as electrostatic interaction, π-π, hydrogen bonding, Yoshida hydrogen bonding, and van der Waals forces, etc [70]. CPH and NPH exhibited higher *K*_*2*_ values, suggesting that the sorption rate was higher than that of PPH. The IPD model was employed to test the sorption-controlling step [71,72]. However, the data indicated that numerous mechanisms are involved in the uptake process.

**Table 3:** Kinetics studies for the pollutants onto pomegranate materials.

Kinetic models	Parameters	PPH			CPH			NPH		
		Cd(II)	IBU	MB	Cd(II)	IBU	MB	Cd(II)	IBU	MB
PFO	*q*_*e*_*(mg/g)*	10.43	3.501	14.60	27.59	8.310	31.46	40.45	15.31	45.30
	*K*_*1*_*(min*^*−*^*^1^)*	0.318	0.221	0.357	0.380	0.255	0.388	0.436	0.385	0.472
	*R*^*2*^	0.849	0.791	0.850	0.883	0.807	0.880	0.895	0.834	0.899
PSO	*q*_*e*_*(mg/g)*	31.30	14.99	39.01	70.99	31.07	79.76	91.05	37.80	93.51
	*K*_*2*_*(g.mg/min)*	0.926	0.918	0.935	0.978	0.956	0.976	0.982	0.951	0.986
	*R*^*2*^	0.990	0.989	0.991	0.993	0.990	0.995	0.991	0.990	0.993
IPD	*C (mg/g)*	14.50	7.405	21.60	28.70	20.41	33.91	41.41	16.80	43.78
	*K*_*i*_*(g/g.min*^*1/2*^*)*	0.419	0.395	0.443	0.446	0.423	0.457	0.529	0.468	0.628
	*R*^*2*^	0.968	0.951	0.960	0.971	0.961	0.979	0.977	0.965	0.983
Experimental	*q*_*e*_*(mg/g)*	32.74	15.80	40.86	72.58	32.49	80.88	92.71	39.76	95.80

#### 4.4.3 Thermodynamic parameters

The thermodynamic parameters *ΔH*^*o*^, *ΔS*^*o*^ and *ΔG*^*o*^ were calculated using equations (6), (7).

(6) $${In\;K}_{c}=-\frac{\Delta H^{o}}{RT}-\frac{{\Delta S}^{o}}{R}$$

(7) $${\Delta G}^{o}=-RT\;In\;K_{c}$$

Temperature dependant parameters such as △*H*^*o*^, △*S*^*o*^, and △*G*^*o*^ were evaluated, and the data is presented in Table 4. The data for the thermodynamic parameter △*H*^*o*^ suggest that all the sorption processes were endothermic; the obtained positive values indicate this [73]. Furthermore, the literature suggests that △*H*^*o*^ values between 20 and 79 kJ/mol indicate that physisorption was involved, while 80–400 kJ/mol shows chemisorption [74]. The △*H*^*o*^ for PPH was between (64.33–69.08 kJ/mol), CPH (82.84–86.03 kJ/mol) and NPH (87.17–88.96 kJ/mol). Therefore, the sorption for PPH was physisorption, and chemisorption for CPH and NPH. The △*S*^*o*^ has positive values, showing increased freedom during the sorption [75]. The △*G*^*o*^ values were negative at all temperatures, demonstrating the reaction's viability. Furthermore, the values obtained for △*G*^*o*^ increased when temperature increased, suggesting that the sorption processes were endothermic [15].

**Table 4:** Thermodynamic studies for the pollutants onto pomegranate materials.

Adsorbents	Temperature (K)	Thermodynamic parameters								
		△H^o^ (KJ/mol)			△S^o^ (KJ/mol)			△Go (J/mol.K)		
		Cd(II)	IBU	MB	Cd(II)	IBU	MB	Cd(II)	IBU	MB
PPH	288	67.69	64.33	69.08	0.3282	0.2101	0.3390	−1.214	−1.081	−1.496
	298							−1.941	−1.756	−2.114
	308							−3.805	−3.346	−3.983
CPH	288	85.38	82.84	86.03	0.4622	0.4524	0.4703	−3.271	−2.884	−3.358
	298							−5.010	−4.373	−5.327
	308							−7.133	−6.958	−7.251
NPH	288	88.21	87.17	88.96	0.4937	0.4612	0.4990	−3.368	−2.971	−3.511
	298							−5.471	−5.014	−5.586
	308							−7.425	−7.001	−7.883

### 4.5 Sorption mechanism

**Scheme 1:**
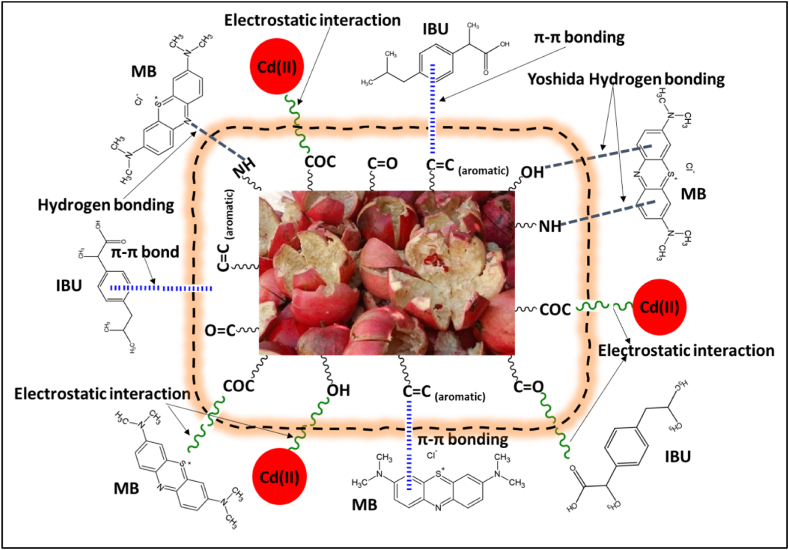
Proposed adsorption mechanism for the sorption of Cd(II), IBU and MB on pomegranate-based sorbents.

Understanding the materials’ uptake mechanism(s) is essential in water treatment applications. In a bid to uncover the mechanism(s), FTIR analysis (Fig. 1) and data for kinetics (Table 3) were utilized to predict the processes involved in the removal of Cd(II), IBU and MB by pomegranate-based adsorbents. FTIR data showed that the materials had oxygen-containing groups on their surfaces; namely, –OH, –C]O, –COC, and as well as –NH and aromatic rings –C]C. In acidic conditions, the functional groups acquired a positive charge; therefore, hydrogen bonding and Yoshida hydrogen bonding were enhanced during the sorption of IBU and MB [50]. Other processes involved π-metal and π-π bonding. On the other hand, in alkaline conditions, the groups acquired a negative charge, thus enhancing the electrostatic interaction between the pollutants and the materials. This agrees with the data for PSO kinetics, which predicted syngenetic mechanisms involving electrostatic interaction, π-π, hydrogen bonding, and van der Waals forces (Scheme 1).

### 4.6 Comparison studies

The sorption capacities obtained for Cd(II), IBU and MB on pomegranate-based materials were compared with previously reported agricultural biosorbents (Table 5). The results obtained for pomegranate husk carbon exhibited higher sorption uptake than some biosorbents. The data suggest that pomegranate-based materials can potentially be employed in water treatment applications to remove numerous pollutants. Data from this study indicate that pomegranate-based materials have potential commercial use.

**Table 5:** Comparison study of sorption capacity (q_e_) of Cd(II), IBU and MB for different biosorbents.

Biosorbent	q_e_ (mg/g)	Pollutant	Reference
Pomegranate husk carbon/Fe(II)–Fe(III) NP	92.75	Cd(II)	This study
Pinecone	92.70		[76]
Barley husk	75.20		[77]
Pomegranate husk activated carbon	68.60		[78]
Modified pomegranate husk	46.29		[79]
Rice husk biochar	17.8		[80]
Activated coconut husk	76.92	IBU	[81]
Activated carbon oak	45.45		[82]
Pomegranate husk carbon/Fe(II)–Fe(III) NP	39.80		This study
Cocoa shell	38.95		[83]
Sugarcane bagasse	13.51		[84]
Olive waste cake	12.90		[85]
Banana peel	250.0	MB	[14]
Watermelon rinds	188.8		[12]
Coconut leaf	112.3		[27]
Pomegranate husk carbon/Fe(II)–Fe(III) NP	95.77		This study
Palmyrah sprout casing	27.67		[86]
Maize stalk pith	7.330		[87]

### 4.7 Post adsorption studies

#### 4.7.1 SEM

**Fig. 10:**
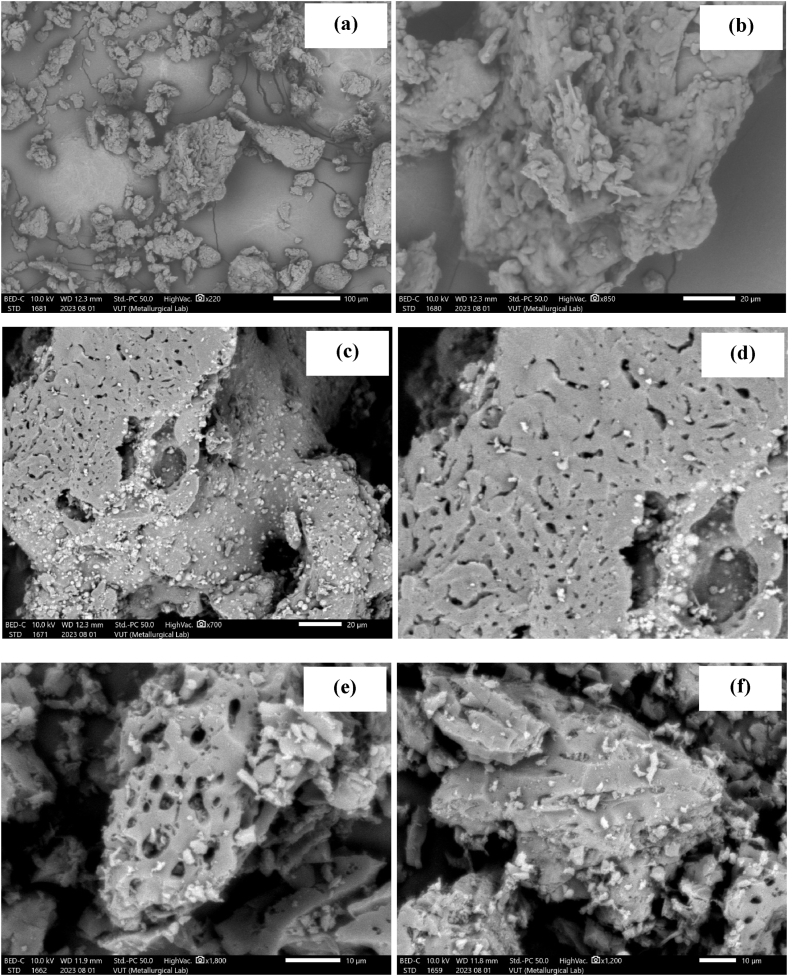
SEM images for PPH (a–b), CPH (c–d), and NPH (e–f).

Post-adsorption studies were carried out on solutions containing the pollutants co-existing in wastewater. The surface of the adsorbents was analyzed after-loading the pollutants onto the materials. The SEM micrographs for PPH in Fig. 10a-b reveal that the surface changed slightly post-adsorption. The images for CPH in Fig. 10c-d and NPH in Fig. 10e-f show that the surface morphology exhibited pores of different sizes. Previous studies investigating plant-based materials show that the pollutants were trapped in the pores, enhanced diffusion [88].

#### 4.7.2 FTIR

The materials post adsorption, loaded with Cd(II), IBU and MB were tested using FTIR. Table 6 recorded slight changes in wavenumbers. The functional groups such as –OH/–NH, –C]C, –C]O, and –COC shifted slightly to lower wavenumbers for all the materials, while –Fe-O on NPH shifted its position to higher wavenumber post adsorption. This is indicative of the involvement of these groups in pollutants uptake. It was observed that the peak for –CH did not change its position; this indicated the non-involvement of the group in the uptake.

**Table 6:** FTIR results before and post sorption.

Functional Group	PPH		CPH		NPH	
	Before sorption	Post sorption	Before sorption	Post sorption	Before sorption	Post sorption
**–OH/–NH**	3207	3190	3232	3198	–	–
**–CH**	–	–	2980	2980	2980	2980
–C]C _(aromatic)_	1596	1590	1596	1588	–	–
	1680	1671	1680	1668	–	–
	1640	1636	1640	1630	–	–
**–C=O**	1400	1388	1400	1396	1400	1389
**–COC**	1042	1040	1042	1037	1042	1035
	1031	1028	1031	1030	1031	1030
	1004	1000	1004	997	1004	995
**–Fe-O**	–	–	–	–	617	620
	–	–	–	–	567	574

## 5 Conclusion

This work developed low-cost pomegranate-based materials for the sorption of a pharmaceutical product (ibuprofen), organic dye (methylene blue), and toxic metal ions of cadmium from aqueous solutions. Carbon from pomegranate husks was obtained at 400 °C, and Fe(II)–Fe(III) nanoparticles were loaded into the surface by co-precipitation. The removal efficiency increased with a progressive increase in pollutant concentrations. The highest adsorption capacity recorded for the materials is 92.80, 39.88 and 95.91 mg/g for NPH, 72.59, 32.55 and 80.58 mg/g for CPH and 32.81, 15.80 and 40.83 mg/g for PPH towards Cd(II), IBU and MB respectively. The data for contact time revealed that the removal efficiency had three steps. Step 1: rapid increase at the initial stage. Step 2: marginal uptake. Step 3: plateau. NPH attained the highest rate, followed by CPH and PPH. pH effect studies revealed that acidic pH solution negatively impacted the removal efficiencies of the pollutants. A solution pH of 2 recorded the lowest removal efficiency. At this condition, the electrostatic interaction was highly restricted, by the protonated (H^+^ or H_3_O^+^) functional groups on the surface of the adsorbents. The best pH value of the solution for the sorption processes was pH 8. The trends showed that sorption was influenced by temperature variation. PSO (*R*^*2*^) values were higher than those for PFO, indicating that PSO described the experimental data better. PSO is based on the chemisorption process, which involves syngenetic mechanisms. The thermodynamic parameter △*H*^*o*^ indicated that all the sorption processes were endothermic. The △*G*^*o*^ possessed negative values demonstrating the reactions viability.

## Author contribution statement

Ntaote David Shooto: Conceived and designed the experiments; Performed the experiments; Analyzed and interpreted the data; Contributed reagents, materials, analysis tools or data; Wrote the paper.

## Data availability statement

Data will be made available on request.

## Declaration of competing interest

The authors declare that they have no known competing financial interests or personal relationships that could have appeared to influence the work reported in this paper.

## References

[1] Nkutha C.S., Naidoo E.B., Shooto N.D. (2021). Adsorptive studies of toxic metals ions of Cr(VI) and Pb(II) from synthetic wastewater by pristine and calcined coral limestones. S. Afr. J. Chem. Eng..

[2] Mabungela N., Shooto N.D., Dikio E.D., Modise S.J., Monapathi M.E., Mtunzi F.M., Xaba T. (2022). Multi-application fennel-based composites for the adsorption of Cr(VI) ions from water and control of Escherichia coli and Staphylococcus aureus. Environmental Chemistry and Ecotoxicology.

[3] Hasan K., Shahriar A., Jim K.U. (2019). Water pollution in Bangladesh and its impact on public health. Heliyon.

[4] Xing T., Wu Y., Wang Q., Sadrnia A., Behmaneshfar A., N Dragoi E. (2023).

[5] Srivastava V., Zare E.N., Makvandi P., Zheng X.-Q., Iftekhar S., Wu A., Padil V.V.T., Mokhtari B., Varma R.S., Tay F.R., Sillanpaa M. (2020). Cytotoxic aquatic pollutants and their removal by nanocomposite-based sorbents. Chemoshere.

[6] Heidari G., Afruzi F.H., Zare E.N. (2023). Molecularly imprinted magnetic nanocomposite based on carboxymethyl dextrin for removal of ciprofloxacin antibiotic from contaminated water. Nanomaterials.

[7] Jan-Roblero J., Cruz-Maya J.A. (2023). Ibuprofen: toxicology and biodegradation of an emerging contaminant. Molecules.

[8] Thabede P.M., Shooto N.D., Naidoo E.B. (2020). Adsorption studies of toxic cadmium(II) and chromium(VI) ions from aqueous solution by activated black cumin (Nigella sativa) seeds. J. Environ. Chem. Eng..

[9] Alsafran M., Saleem M.H., Jabri H.A., Rizwan M., Usman K. (2023). Principles and applicability of integrated remediation strategies for heavy metal removal/recovery from contaminated environments. J. Plant Growth Regul..

[10] Shooto N.D., Thabede P.M., Bhila B., Moloto H., Naidoo E.B. (2020). Lead ions and methylene blue dye removal from aqueous solution by mucuna beans (velvet beans) adsorbents. J. Environ. Chem. Eng..

[11] Thabede P.M., Shooto N.D., Naidoo E.B. (2020). Removal of methylene blue dye and lead ions from aqueous solution using activated carbon from black cumin seeds. S. Afr. J. Chem. Eng..

[12] Jawad A.H., Ngoh Y.S., Radzun K.A. (2020). Utilization of watermelon (Citrullus lanatus) rinds as a natural low-cost biosorbent for adsorption of methylene blue: kinetic, equilibrium and thermodynamic studies. J. Taibah Univ. Sci..

[13] Al-Tohamy R., Ali S.S., Li F., Okasha K.M., Mahmoud Y.A.-G., Elsamahy T., Jiao H., Fu Y., Sun J. (2022). A critical review on the treatment of dye-containing wastewater: ecotoxicological and health concerns of textile dyes and possible remediation approaches for environmental safety. Ecotoxicol. Environ. Saf..

[14] Jawad A.H., Rashid R.A., Ishak M.A.M., Ismail K. (2018). Adsorptive removal of methylene blue by chemically treated cellulosic waste banana (*Musa sapientum*) peels. J. Taibah Univ. Sci..

[15] Lellis B., Favaro-Polonio C.Z., Pamphile J.A., Polonio J.C. (2019). Effects of textile dyes on health and the environment and bioremediation potential of living organisms. Biotechnology Research and Innovation.

[16] Ternes T.A., Meisenheimer M., Mcdowell D., Sacher F., Brauch H.-J., Haist-Gulde B. (2002). Removal of pharmaceuticals during drinking water treatment. Environ. Sci. Technol..

[17] Reghioua A., Barkat D., Jawad A.H., Abdulhameed A.S., Rangabhashiyam S., Khan M.R., Alothman Z.A. (2021). Magnetic chitosan-glutaraldehyde/zinc oxide/Fe_3_O_4_ nanocomposite: optimization and adsorptive mechanism of remazol brilliant blue R dye removal. J. Polym. Environ..

[18] Mabungela N., Shooto N.D., Mtunzi F., Naidoo E.B. (2022). The adsorption of copper, lead metal ions, and methylene blue dye from aqueous solution by pristine and treated fennel seeds. Adsorpt. Sci. Technol..

[19] Higgins P., Siddiqui S.H., Kumar R. (2022). Design of novel graphene oxide/halloysite nanotube@polyaniline nanohybrid for the removal of diclofenac sodium from aqueous solution. Environ. Nanotechnol. Monit. Manag..

[20] Aldahash S.A., Higgins P., Siddiqui S.H., Uddin M.K. (2022). Fabrication of polyamide-12/cement nanocomposite and its testing for different dyes removal from aqueous solution: characterization, adsorption, and regeneration studies. Sci. Rep..

[21] Hassanzadeh-Afruzi F., Esmailzadeh F., Heidari G., Maleki A., Zare E.N. (2023). Arabic gum-grafted-hydrolyzed polyacrylonitrile@ZnFe2O4 as a magnetic adsorbent for remediation of levofloxacin antibiotic from aqueous solutions. ACS Omega.

[22] Alluhaybi A.A., Hameed A.M., Alotaibi M.T., Alharbi A., Shahat A. (2023). Synthesis and characterization of carbon nanospheres for adsorption of ibuprofen from aqueous solution: optimization by Box–Behnken design. J. Mol. Liq..

[23] Mabungela N., Shooto N.D., Mtunzi F., Naidoo E.B., Mlambo M., Mokubung K.E., Mpelane S. (2023). Multi-application of fennel (Foeniculum vulgaris) seed composites for the adsorption and photo-degradation of methylene blue in water. S. Afr. J. Chem. Eng..

[24] Thabede P.M., Shooto N.D., Xaba T., Naidoo E.B. (2020). Sulfuric activated carbon of black cumin (nigella sativa L.) seeds for the removal of cadmium(II) and methylene blue dye. Asian J. Chem..

[25] Mabungela N., Shooto N.D., Mtunzi F., Naidoo E.B. (2022).

[26] Jawad A.H., Bardhan M., Islam A., Islam A., Syed-Hassan S.S.A., Surip S.N. (2020). Insights into the modeling, characterization and adsorption performance of mesoporous activated carbon from corn cob residue via microwave- assisted H_3_PO_4_ activation. Surface. Interfac..

[27] Jawad A.H., Rashid R.A., Mahmuod R.M.A., Ishak M.A.M., Kasim N.N., Ismai K. (2015). Adsorption of methylene blue onto coconut (Cocos nucifera) leaf: optimization, isotherm and kinetic studies. Desalination Water Treat..

[28] Barnossi A.E., Moussaid F., Housseini A.I. (2021). Tangerine, banana and pomegranate peels valorisation for sustainable environment: a review. Biotechnology Reports.

[29] Opara I.K., Fawole O.A., Opara U.L. (2021). Postharvest losses of pomegranate fruit at the packhouse and implications for sustainability indicators. Sustainability.

[30] Ghaedi A.M., Ghaedi M., Vafaei A., Iravani N., Keshavarz M., Rad M., Tyadi I., Agarwal S., Gupta V.K. (2015). Adsorption of copper(II) using modified activated carbon prepared from pomegranate wood: optimization by bee algorithm and response surface methodology. J. Mol. Liq..

[31] Ben-Ali S., Jaoali I., Souissi-Najar, Ouedermi A. (2017). Characterization and adsorption capacity of raw pomegranate peel biosorbents for copper. J. Clean. Prod..

[32] Ahmad M.A., Eusoff M.A., Oladoye P.O., Adegoke K.A., Bello O.S. (2020). Statistical optimization of Remazol brilliant blue R dye adsorption onto activated carbon prepared from pomegranate fruit peel. Chemical Data Collections.

[33] Rashtbari Y., Hazrati S., Azari A., Afshin S., Fazlzadeh M., Vosoughi M. (2020). A novel, eco-friendly and green synthesis of PPAC-ZnO and PPAC-nZVI nanocomposite using pomegranate peel: cephalexin adsorption experiments, mechanisms, isotherms and kinetics. Adv. Powder Technol..

[34] Hadi S., Taheri E., Amin M.M., Fatehizadeh A., Aminabhavi T.M. (2021). Adsorption of 4-chlorophenol by magnetized activated carbon from pomegranate husk using dual stage chemical activation. Chemosphere.

[35] Ahmad M.A., Eusoff M.A., Oladoye P.O., Adegoke K.A., Bello O.S. (2021). Optimization and batch studies on adsorption of methylene blue dye using pomegranate fruit pee; based adsorbent. Chemical Data Collections.

[36] Ramadan H.S., Mobarak M., Lima E.C., Bonilla-Petriciolet A., Li Z., Seliem M.K. (2021). Cr(VI) adsorption onto a new composite prepared from Meidum black clay and pomegranate peel extract: experiments and physicochemical interpretations. J. Environ. Chem. Eng..

[37] Suhaimi A., Abdulhameed A.S., Jawad A.H., Yousef T.A., Al Duaij O.K., Alothman Z.A., Wilson L.D. (2022). Production of large surface area activated carbon from a mixture of carrot juice pulp and pomegranate peel using microwave radiation-assisted ZnCl_2_ activation: an optimized removal process and tailored adsorption mechanism of crystal violet dye. Diam. Relat. Mater..

[38] Akkari I., Spessato L., Graba Z., Bezzi N., Kaci M.M. (2023). A sustainably produced hydrochar from pomegranate peels for the purification of textile contaminants in an aqueous medium. Sustainable Chemistry and Pharmacy.

[39] Shooto N.D., Thabede P.M. (2022). Binary adsorption study of Chromium and Cadmium metal ions from hemp (Cannabis sativa) seeds based adsorbents. Environ. Nanotechnol. Monit. Manag..

[40] Tomczyk A., Sokołowska Z., Boguta P. (2020). Biochar physicochemical properties: pyrolysis temperature and feedstock kind effects. Rev. Environ. Sci. Biotechnol..

[41] Mopoung S., Dejang N. (2021). Activated carbon preparation from eucalyptus wood chips using continuous carbonization–steam activation process in a batch intermittent rotary kiln. Sci. Rep..

[42] Jawad A.H., Ishak M.A.M., Farhan A.M., Ismail K. (2017). Response surface methodology approach for optimization of colour removal and COD reduction of methylene blue using microwave-induced NaOH activated carbon from biomass waste. Desalination Water Treat..

[43] Raghubanshi H., Ngobeni S.M., Osikoya A.O., Shooto N.D., Dikio C.W., Naidoo E.B., Dikio E.D. (2017). Synthesis of graphene oxide and its application for the adsorption of Pb^2+^ from aqueous solution. J. Ind. Eng. Chem..

[44] Jawad A.H., Abdulhameed A.S., Hanafiah M.A.K.M., Alothman Z.A., Khan M.R., Surip S.N. (2021). Numerical desirability function for adsorption of methylene blue dye by sulfonated pomegranate peel biochar: modeling, kinetic, isotherm, thermodynamic, and mechanism study. Kor. J. Chem. Eng..

[45] Jawad A.H., Waheeb A.S., Rashid R.A., Nawawi W.I., Yousif E. (2018). Equilibrium isotherms, kinetics, and thermodynamics studies of methylene blue adsorption on pomegranate (*Punica granatum*) peels as a natural low-cost biosorbents. Desalination Water Treat..

[46] D Shooto N. (2020). Removal of toxic hexavalent chromium (Cr(VI)) and divalent lead (Pb(II)) ions from aqueous solution by modified rhizomes of Acorus calamus. Surface. Interfac..

[47] Giri R., Kumari N., Behera M., Sharma A., Kumar S., Kumar N., Singh R. (2021). Adsorption of hexavalent chromium from aqueous solution using pomegranate peel as low-cost biosorbent. Environmental Sustainability.

[48] Shooto N.D. (2020). Removal of lead(II) and chromium(VI) ions from synthetic wastewater by the roots of harpagophytum procumbens plant. J. Environ. Chem. Eng..

[49] Thabede P.M., Shooto N.D., Xaba T., Naidoo E.B. (2021). Magnetite functionalized Nigella Sativa seeds for the uptake of chromium(VI) and lead(II) ions from synthetic wastewater. Adsorpt. Sci. Technol..

[50] Jawad A.H., Abdulhameed S.A., Bahrudin N.N., Hum N.N.M.F., Surip N.S., Syed-Hassan S.S.A., Yousif E., Sabar S. (2021). Microporous activated carbon developed from KOH activated biomass waste: surface mechanistic study of methylene blue dye adsorption. Water Sci. Technol..

[51] Ishak W.H.W., Ahmad I., Ramli S., Amin M.C.I.M. (2018). Gamma irradiation-assisted synthesis of cellulose nanocrystal-reinforced gelatin hydrogels. Nanomaterials.

[52] Patra J.K., Baek K.-H. (2017). Green biosynthesis of magnetic iron oxide (Fe_3_O_4_) nanoparticles using the aqueous extracts of food processing wastes under photo-catalyzed condition and investigation of their antimicrobial and antioxidant activity. J. Photochem. Photobiol. B Biol..

[53] Shooto N.D., Naidoo E.B., Maubane M. (2019). Sorption studies of toxic cations on ginger root adsorbent. J. Ind. Eng. Chem..

[54] Nkutha C.S., Shooto N.D., Naidoo E.B. (2020). Adsorption studies of methylene blue and lead ions from aqueous solution by using mesoporous coral limestones. S. Afr. J. Chem. Eng..

[55] Diagboya P.N., Dikio E.D. (2018). Scavenging of aqueous toxic organic and inorganic cations using novel facile magneto-carbon black-clay composite adsorbent. J. Clean. Prod..

[56] Shooto N.D., Dikio E.D. (2018). Highly porous MOF adsorbent for wastewater treatment. Asian J. Chem..

[57] Phuonga D.T.M., Loca N.X., Miyanishi T. (2019). Efficiency of dye adsorption by biochars produced from residues of two rice varieties, Japanese Koshihikari and Vietnamese IR50404. Desalination Water Treat..

[58] Yang X., Wan Y., Zheng Y., He F., Yu Z., Huang J., Wang H., Ok Y.S., Jiang Y., Gao B. (2019). Surface functional groups of carbon-based adsorbents and their roles in the removal of heavy metals from aqueous solutions: a critical review. Chem. Eng. J..

[59] Riyad Y.M., Elmorsi T.M., Alam M.G., Abel B. (2023). Surface functionalization of bioactive hybrid adsorbents for enhanced adsorption of organic dyes. Int. J. Environ. Res. Publ. Health.

[60] Ahmad F.A. (2023). The use of agro-waste-based adsorbents as sustainable, renewable, and low-cost alternatives for the removal of ibuprofen and carbamazepine from water. Heliyon.

[61] Hou F., Wang D., Ma X., Fan L., Ding T., Ye X., Liu D. (2021). Enhanced adsorption of Congo red using chitin suspension after sonoenzymolysis. Ultrason. Sonochem..

[62] Langmuir I. (1916). The constitution and fundamental properties of solids and liquids. J. Am. Chem. Soc..

[63] Freundlich H.M.F. (1906). Uber die adsorption in losungen. Z. Phys. Chem..

[64] Bashiri H., Orouji S. (2015). A new isotherm for multilayer gas adsorption on heterogeneous solid surfaces. Theor. Chem. Acc..

[65] Ayawei N., Ebelegi A.N., Wankasi D. (2017). Modelling and interpretation of adsorption isotherms. J. Chem..

[66] Pimentel C.H., Freire M.S., Gómez-Díaz D., González-Álvarez D. (2023). Removal of wood dyes from aqueous solutions by sorption on untreated pine (Pinus radiata) sawdust. Cellulose.

[67] Siddiqui S.I., Chaudhry S.A. (2019). Nanohybrid composite Fe_2_O_3_-ZrO_2_/BC for inhibiting the growth of bacteria and adsorptive removal of arsenic and dyes from water. J. Clean. Prod..

[68] To H.-H., Hadi P., Hui C.-W., Lin C.S.L., McKay G. (2017). Mechanism study of atenolol, acebutolol and carbamazepine adsorption on waste biomass derived activated carbon. J. Mol. Liq..

[69] Shikuku V.O., Jemutai-Kimosop S. (2020). Efficient removal of sulfamethoxazole onto sugarcane bagasse-derived biochar: two and three-parameter isotherms, kinetics and thermodynamics. S. Afr. J. Chem..

[70] Saggu M., Levinson N.M., Boxer S.G. (2012). Experimental quantification of electrostatics in X-H···π hydrogen bonds. J. Am. Chem. Soc..

[71] Wang J., Guo X. (2022). Rethinking of the intraparticle diffusion adsorption kinetics model: interpretation, solving methods and applications. Chemosphere.

[72] Al-Odayni A.-B., Alsubaie F.S., Saeed W.S. (2023). Nitrogen-rich polyaniline-based activated carbon for water treatment: adsorption kinetics of anionic dye methyl orange. Polymers.

[73] Tutu H., Bakatula E., Dlamini S., Rosenberg E., Kailasam V., Cukrowska E.M. (2013). Kinetic, equilibrium and thermodynamic modelling of the sorption of metals from aqueous solution by a silica polyamine composite. WaterSA.

[74] Shooto N.D., Dikio E.D., Wankasi D., Sikhwivhilu L.M. (2017). Iron based metal organic framework as an effective lead ions remover from aqueous solution: thermodynamic and kinetics studies. Hemjska Industrija.

[75] Shooto N.D., Ayawei N., Wankasi D., Sikhwivhilu L., Dikio E.D. (2016). Study of cobalt metal organic framework material as adsorbent for lead ions removal in aqueous solution. Asian J. Chem..

[76] Balarak D., Azarpira H., Mostapour F.K. (2016). Thermodynamics of removal of cadmium by adsorption on barley husk biomass. Der Pharma Chem..

[77] Teng D., Zhang B., Xu G., Wang B., Mao K., Wang J., Sun J., Feng X., Yang Z., Zhang H. (2020). Efficient removal of Cd(II) from aqueous solution by pinecone biochar: sorption performance and governing mechanisms. Environ. Pollut..

[78] Al-Onazi W.A., Ali M.H.H., Al-Garni T. (2021). Using pomegranate peel and date pit activated carbon for the removal of cadmium and lead ions from aqueous solution. J. Chem..

[79] Wang F., Wu P., Shu L., Guo Q., Huang D., Lui H., Isotherm (2021). Kinetics and adsorption mechanism studies of diethylenetriaminepentaacetic acid-modified banana/pomegranate peels as efficient adsorbents for removing Cd(II) and Ni(II) from aqueous solution. Environ. Sci. Pollut. Res..

[80] Saeed A.A.H., Harun N.Y., Nasef M.M., Al-Fakih A., Ghaleb A.A.S., Afolabi H.K. (2022). Removal of cadmium from aqueous solution by optimized rice husk biochar using response surface methodology. Ain Shams Eng. J..

[81] Bello O.S., Moshood M.A., Ewetumo B.A., Afolabi I.C. (2020). Ibuprofen removal using coconut husk activated biomass. Chemical Data Collections.

[82] Nourmoradi H., Moghadam K.F., Jafari A., Kamarehie B. (2018). Removal of acetaminophen and ibuprofen from aqueous solutions by activated carbon derived from quercus brantii (oak) acorn as a low-cost biosorbent. J. Environ. Chem. Eng..

[83] Al-Yousef H., Alotaibi B.M., Aouaini F., Sellaoui L., Bonilla-Petriciolet A. (2021). Adsoption of ibuprofen on cocoa shell biomass-based adsorbents: interpretation of the adsorption equilibrium via statistical physics theory. J. Mol. Liq..

[84] Chakraborty P., Show S., Banerjee S., Halder G. (2018). Mechanistic insight into sorptive elimination of ibuprofen employing bidirectional activated biochar from sugarcane bagasse: performance evaluation and cost estimation. J. Environ. Chem. Eng..

[85] Dubeya S.P., Dwivedi A.D., Sillanpaaa M., Gopal K. (2010). *Artemisia vulgaris*-derived mesoporous honeycomb-shaped activated carbon for ibuprofen adsorption. Chem. Eng. J..

[86] Jayasuriya D.M.N.H., Nadarajah K. (2023). Understanding association between methylene blue dye and biosorbent: palmyrah sprout casing in adsorption process in aqueous phase. Water Sci. Eng..

[87] Li J., Tang X., Zhang H., Gao X., Zhang S., Tan T. (2022). Adsorption behaviour of three-dimensional bio-adsorbent from maize stalk pith for methylene blue. Ind. Crop. Prod..

[88] Mitic-Stojanovic D.-L., Zarubica A., Purenovic M., Bojic D., Andjelkovic T., Bojic A.L. (2011). Biosorptive removal of Pb^2+^, Cd^2+^ and Zn^2+^ ions from water by Lagenaria vulgaris shell. WaterSA.

